# Modelling the transmission of healthcare associated infections: a systematic review

**DOI:** 10.1186/1471-2334-13-294

**Published:** 2013-06-28

**Authors:** Esther van Kleef, Julie V Robotham, Mark Jit, Sarah R Deeny, William J Edmunds

**Affiliations:** 1 Infectious Disease Epidemiology Department, Faculty of Epidemiology and Population Health, Centre of Mathematical Modelling, London School of Hygiene and Tropical Medicine, London, UK; 2 Modelling and Economics unit, Public Health England, Colindale, London, UK

**Keywords:** Mathematical modelling, Healthcare-associated infections, Epidemiology

## Abstract

**Background:**

Dynamic transmission models are increasingly being used to improve our understanding of the epidemiology of healthcare-associated infections (HCAI). However, there has been no recent comprehensive review of this emerging field. This paper summarises how mathematical models have informed the field of HCAI and how methods have developed over time.

**Methods:**

MEDLINE, EMBASE, Scopus, CINAHL plus and Global Health databases were systematically searched for dynamic mathematical models of HCAI transmission and/or the dynamics of antimicrobial resistance in healthcare settings.

**Results:**

In total, 96 papers met the eligibility criteria. The main research themes considered were evaluation of infection control effectiveness (64%), variability in transmission routes (7%), the impact of movement patterns between healthcare institutes (5%), the development of antimicrobial resistance (3%), and strain competitiveness or co-colonisation with different strains (3%). Methicillin-resistant *Staphylococcus aureus* was the most commonly modelled HCAI (34%), followed by vancomycin resistant enterococci (16%). Other common HCAIs, e.g. *Clostridum difficile,* were rarely investigated (3%). Very few models have been published on HCAI from low or middle-income countries.

The first HCAI model has looked at antimicrobial resistance in hospital settings using compartmental deterministic approaches. Stochastic models (which include the role of chance in the transmission process) are becoming increasingly common. Model calibration (inference of unknown parameters by fitting models to data) and sensitivity analysis are comparatively uncommon, occurring in 35% and 36% of studies respectively, but their application is increasing. Only 5% of models compared their predictions to external data.

**Conclusions:**

Transmission models have been used to understand complex systems and to predict the impact of control policies. Methods have generally improved, with an increased use of stochastic models, and more advanced methods for formal model fitting and sensitivity analyses. Insights gained from these models could be broadened to a wider range of pathogens and settings. Improvements in the availability of data and statistical methods could enhance the predictive ability of models.

## Background

Healthcare-associated infections (HCAI) continue to cause a major burden on society, affecting more than 4 million patients annually in Europe alone, and causing an estimated 16 million additional bed-days responsible for €7 billion in direct medical costs [1]. In the United Kingdom, interventions such as improved hand hygiene, antibiotic stewardship and screening combined with decolonisation are believed to have set off a steep reduction in reported incidence of health care-associated methicillin-resistant *Staphylococcus aureus* (MRSA) bacteraemia and *Clostridium difficile* infection with peak incidence in 2003/04 and 2007/08 respectively [2]. Further progress in reducing the burden of HCAI is hindered by uncertainty surrounding the role of asymptomatic carriers [3,4], environmental transmission [5-7] and the recent emergence of bacteria other than MRSA and *C. difficile*, such as enterobacteriaceae (e.g. *Escherichia coli*) [8]*.* Mathematical models are increasingly being used to obtain a deeper understanding of epidemiological patterns in hospital infections and to guide hospital infection control policy decisions, as is seen in other areas of infectious disease epidemiology [9].

A previous review of the area provided insight into the type of models used for hospital epidemiology and highlighted their capacity to increase epidemiological understanding, and inform infection control policy [10]. This review, conducted in 2006, primarily aimed to explain the capacities of models and therefore was limited to a detailed description of a number of studies. Hence, the emerging trends in the area were not fully explored. Since 2006 the field has expanded considerably. We conducted a systematic review in order to establish how mathematical models have been applied in the field of HCAI, and how model methods have developed over time.

## Methods

We searched Medline (1950 to present), EMBASE (1947 to present), Scopus (1823 to present), CINAHL (1937 to present) and Global health (1910 to present). Results were limited to peer-reviewed publications in English. Search terms and Medical Subject Headings (MeSH) for nosocomial organisms and antibiotic resistance were combined with search and MeSH terms for healthcare settings and mathematical models as follows:

•Nosocomial infections in general (e.g.”*healthcare-associated infection$”* or *“hospital-acquired infection$”*)

OR

•Nosocomial organisms (e.g. *“C. difficile” or “Staphylococcus aureus”)***OR** Antimicrobial resistance **AND** Nosocomial (e.g. *“hospital$” or “healthcare”*)

AND

•Mathematical modelling or economic evaluation model (e.g. *“stochastic” or “deterministic”***AND***“model”)*

We decided not to use search terms for nosocomial infection types (e.g. surgical site infections or urinary tract infections), since our review focuses on the transmission of infections from one individual to another, which cannot generally be accurately represented without knowing the causative organism.

The complete search strategy is provided in the Additional file 1. All databases were search identically, with exception of the MeSH terms, which were altered to the subject-heading dictionary used in each particular database. The final search was conducted on 11 December 2011. Each title and abstract in the search result was independently screened by EvK and at least one of the other authors. Full text evaluation was conducted by EvK and in case of uncertainty, discussion took place with JR.

### Inclusion criteria

Eligible studies had to fulfil the following criteria: 1) mathematical modelling of HCAI transmission and/or the dynamics of antimicrobial resistance; 2) dynamic transmission models only (i.e. a model which tracks the number of individuals (or proportion of a population) carrying or infected with a pathogen over time, while capturing the effect of contact between individuals on transmission [9]); 3) a primary focus on HCAI transmission in healthcare settings.

### Exclusion criteria

Studies were excluded if they did not involve: 1) human to human transmission; or did involve 2) within host transmission only; 3) pharmacodynamics and pharmacokinetics of drugs (e.g. the impact of antibiotic exposure, exploring antibiotic tolerance and investigating fitness), 4) animal transmission of HCAI; 5) community transmission of pathogens spread in the healthcare environment as well, where community spread was the focus of the paper (e.g. SARS epidemics); or 6) literature review without new primary studies. Moreover, no editorials or letters to editors were included, except if a new mathematical model was introduced.

## Results

**Figure 1:**
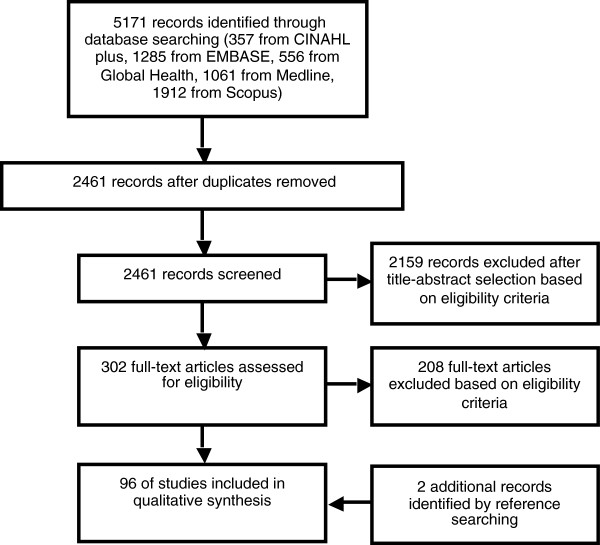
PRISMA flowchart.

The database search retrieved 2461 unique papers (Figure 1). After screening the titles and abstracts, 302 papers met the inclusion criteria and were thus eligible for full text evaluation. Review of the full text publications resulted in the inclusion of 94 relevant papers based on our selection criteria. An additional two papers were identified via reference screening [11,12].

**Figure 2:**
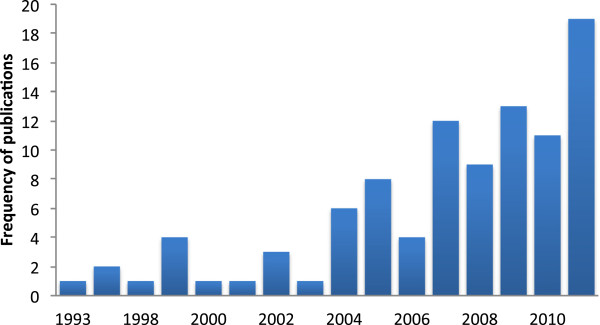
**Number of HCAI modelling publications over time (1993–2011).** Number of studies identified on modelling of HCAI and antimicrobial resistance spread in a nosocomial setting according to year of publication.

The distribution of these 96 papers over time demonstrates that HCAI transmission models have been increasingly employed since the introduction of the first model of nosocomial pathogens’ spread [13] (Figure 2)**.**

### Objectives of mathematical models of HCAIs

#### Pathogens modelled

Although HCAIs are often associated with antibiotic-resistant bacteria, HCAI models have involved antimicrobial susceptible pathogens as well. In this review, studies that did not specify a particular pathogen of concern, but that claimed to investigate antimicrobial resistant bacteria, were classified as antimicrobial resistant bacteria (ARB). Otherwise, the study was categorised as ‘HCAI in general’. Moreover, as the majority of patients can carry HCAI such as MRSA and *C. difficile* asymptomatically, many mathematical models simulate the epidemiology of colonisation, however for brevity we have referred to all models as concerning the epidemiology of HCAI in the text.

**Figure 3:**
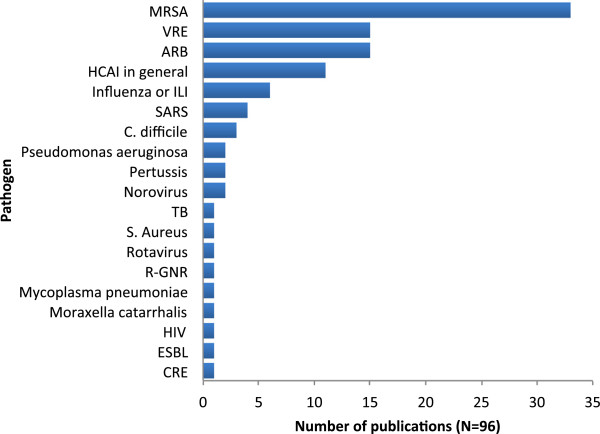
**Pathogens modelled in a nosocomial setting (1993–2011).** Number of studies identified on nosocomial infection transmission according to pathogen type. MRSA= Methicillin resistant Staphylococcus aureus; ARB = Antimicrobial resistant bacteria; VRE = Vancomycin-resistant *Enterococcus*; HCAI = Healthcare associated infections; ILI = Influenza-like illness; SARS = Severe acute respiratory syndrome; TB= Tuberculosis; R-GNR= Third generation cephalosporin-resistant Gram-negative rods; HIV = Human immunodeficiency virus; ESBL = Extended-Spectrum Beta-Lactamases; CRE = cephalosporin-resistant Enterobacteriaceae.

Figure 3 shows that MRSA was the most common bacterial species studied (34%; 33 studies) [14-46], followed by Vancomycin-resistant *Enterococcus* (VRE) (or glycopeptide-resistant enterococci) (16%; 15 studies) [12,18,28,31,47-57] whereas *C. difficile* has rarely been the subject of a model (3%; 3 studies) [58-60]. As several studies investigated the dynamics of more than one pathogen, the total number of infection agents (N=102) listed in Figure 3 exceeds the total number of studies (N=96).

#### Intervention effectiveness

**Figure 4:**
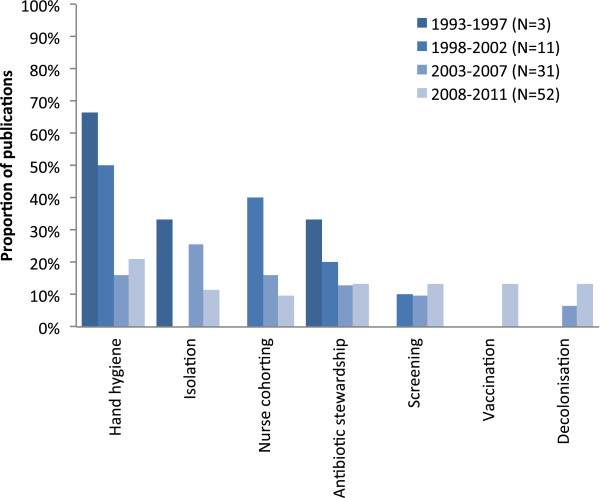
**Main interventions evaluated over time (1993–2011).** Main interventions evaluated over time (1993–2011)**.** Illustration of the proportionate distribution of the seven most commonly investigated interventions by means of a modelling framework by the total number of publications in each time period.

The first model of HCAI conceptualised the spread of antibiotic resistance in bacterial populations among hospital patients [13]. This was soon followed by models evaluating the effectiveness of interventions to reduce antibiotic resistance (e.g. antibiotic cycling or mixing). Since then, most HCAI models have aimed to quantify infection control effectiveness (64%; 62 studies). The infection control measures most frequently considered among these 62 papers have been: hand hygiene (37%; 23 studies), patient isolation (24%; 15 studies), HCW cohorting (23%; 14 studies), antibiotic stewardship (21%; 13 studies), and screening (18%, 11 studies). Figure 4 provides an overview of the main interventions modelled over time, emphasising that decolonisation and vaccination are more recent subjects of study. Moreover, a wider variability of interventions has been evaluated in the later years. Table 1 illustrates the type of interventions that have been evaluated for each HCAI pathogen.

**Table 1:** Definitions of modelling terms

**Term**	**Definition**
**Deterministic model**	A model in which there is no role of chance in the evolution of the states of the system, i.e. the model is ‘predetermined’ by the parameters and initial conditions [61].
**Stochastic model**	A model in which random (stochastic) processes can affect whether certain events or processes occur (e.g. the rate at which individuals are infected can vary by chance) [61].
**Compartmental model**	A model in which the population is divided into subgroups (i.e. compartments), which represent the average values of individuals in a particular state (e.g. susceptible, infectious or recovered). Within each compartment, all individuals are homogenous [9].
**Individual-based model**	A model in which single individuals are tracked rather than subgroups. Hence, each individual can be assigned different characteristics such as the probability of acquiring infection or causing transmission [9].
**Model fitting/ model calibration**	The inference of unknown parameters by choosing their values in order to approximate a set of data as well as possible. Examples of model fitting methods are least squares approximation maximum likelihood estimation and Markov Chain Monte Carlo Methods [62].
**Model validation**	Comparison of model predictions to external data, that is a model should be validated against observations from alternative data to the data used for model fitting [62].
**Univariate sensitivity analysis**	Investigation of uncertainty in model parameters and its impact on model predictions by means of altering one parameter at a time whilst holding others at their base-case value.
**Bi/ multivariate sensitivity analysis**	Investigation of uncertainty in model parameters by means of alteration of two (or more) parameters at a time whilst holding others at their base-case value.
**Probabilistic sensitivity analysis**	A type of multivariate sensitivity analysis where multiple runs of the model are performed with random selection of input parameters.
**Dynamic transmission model**	A model which tracks the number of individuals (or proportion of a population) carrying or infected with a pathogen over time, where the risk of transmission to susceptible at a given point in time is dependent on the number of infected (or colonised) individuals in the community [9].
**Static model**	A model where the transmission risk is treated as a parameter exogenous to the model, i.e. it does not change with the number of infectious individuals in the population [9].
**Force of infection**	The rate at which infected individuals become infected per unit time [61]

#### Furthering epidemiological understanding

Models are often used to increase epidemiological understanding. Hospital surveillance data, which is frequently used to inform HCAI models, can lack detail in what is needed for modelling purposes. For example, information on asymptomatic carriage and timing of events (e.g. infection) are often lacking. Several studies use new statistical methods to overcome such difficulties [31,36,48] and to allow for estimation of important epidemiological parameters (e.g. transmission rates) from different data sources, varying from routinely collected hospital data [56,57] to strain typing [63] or genotype data [64]. Others use modelling techniques to determine the relative importance of potential transmission reservoirs or acquisition routes (of *C. difficile*[58,60], VRE [50,53], cephalosporin-resistant Enterobacteriaceae (CRE) [65] and SARS [66].

The ecological dynamics of pathogens have also been explored using models, including antimicrobial resistance [13,67,68]; co-colonisation with different pathogen strain types [27,46] and competition between strains [24]*.* Another more recent subject of study is the potential impact of readmission of patients from settings such as long-term care facilities (LTCFs) or the community, as well as general movement patterns between healthcare institutes and/or the community on the transmission of MRSA [19,25,38,69], antimicrobial resistance [70] and HCAI in general [71].

Economic outcomes were not considered in dynamic transmission models until 2011 [14,23,72]. Three recent papers applied dynamic modelling techniques to estimate the economic burden of disease (MRSA) [22] and norovirus [69], and to investigate economic incentives for infection control investments [73].

#### Country of study

A number of studies (36%, 32 studies) did not specify a particular national setting. Of the publications that did; only three studies (3%) explored transmission of HCAI in lower and lower middle income countries [22,74,75] and another three looked at upper middle income China [15,66,76]. Studies have mainly concentrated on the United States (16%; 15 studies), the United Kingdom (13%; 12 studies) and the Netherlands (10%; 10 studies).

### Methods employed for mathematical modelling of HCAIs

#### Stochastic vs. deterministic

**Figure 5:**
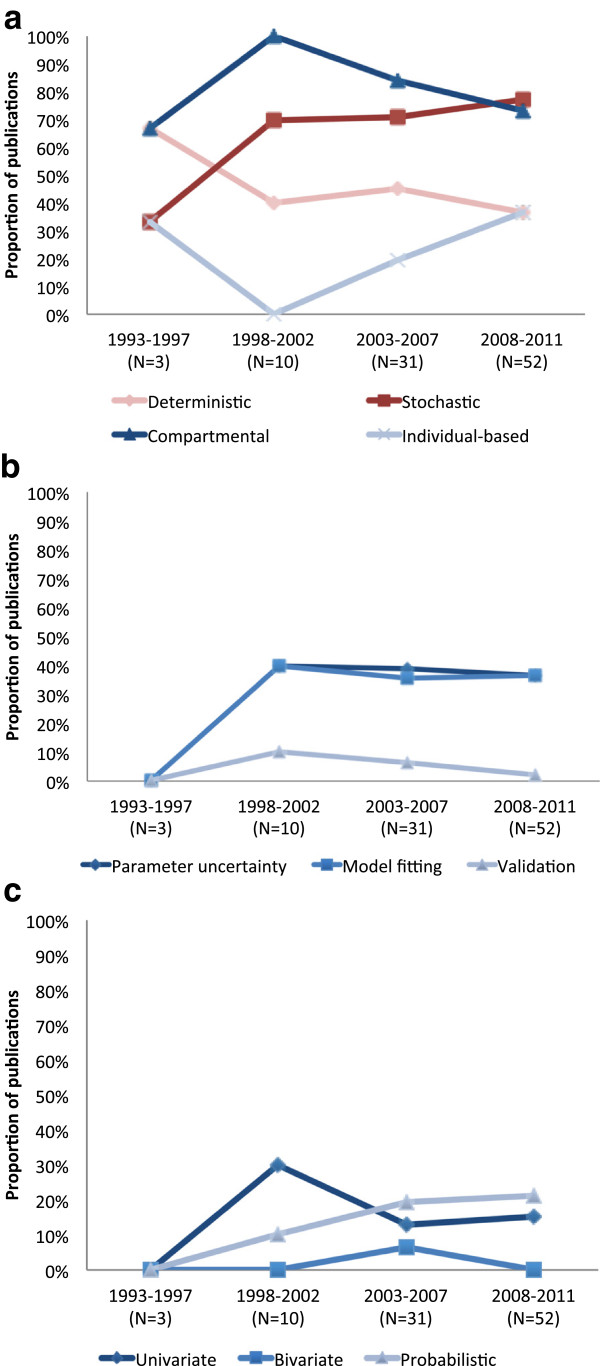
**Development of HCAI model methods used over time (1993–2011).** Application of key modelling characteristics and development over time. *Figure*5*a: Model approach* Proportion of models using a deterministic vs. stochastic and a compartmental vs individual-based modelling approach by the total number of publications in each time period. Note that the categories are not exclusive, i.e. whereas all individual-based models identified are stochastic, compartmental models may be deterministic or stochastic. Moreover, a proportion of studies use a combination of the above listed modelling approaches (e.g. a deterministic compartmental model complemented by a stochastic individual-based model). *Figure*5*b Model methods* Proportion of models that are fitted to data, have included uncertainty and are validated by consultation of two different datasets by total number of publications in each time period. *Figure*5*c Methods used for characterising parameter uncertainty:* Proportion of models that have employed uni-variate, vs bi-variate vs probabilistic sensitivity analysis by total number of publications that incorporated parameter uncertainty in each time period.

**Figure 6:**
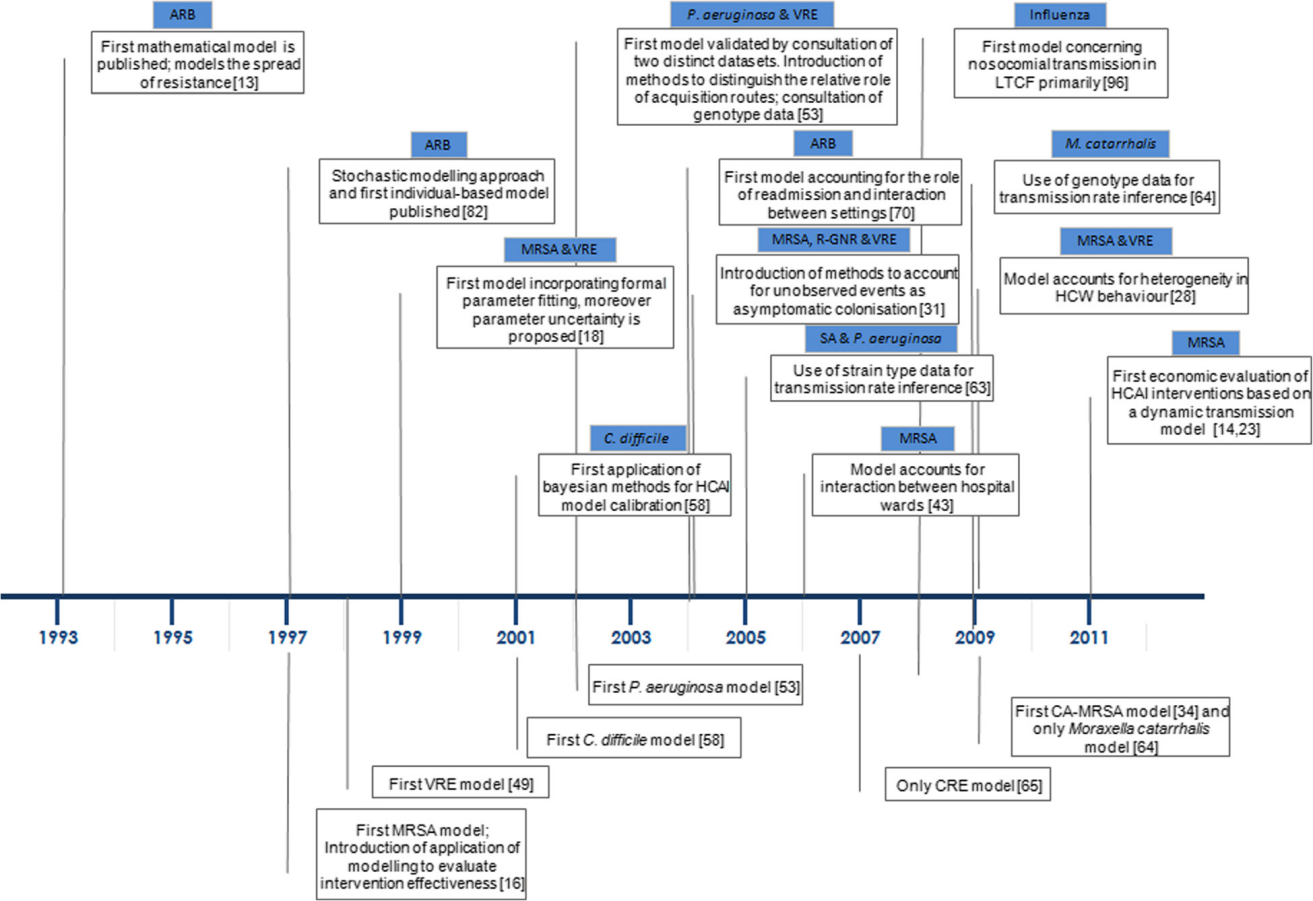
**Milestones of HCAI modelling.** Timeline listing new applications of mathematical models for HCAI and antimicrobial resistance over time as well as improvements of these models according to year of publication.

The first HCAI models captured transmission dynamics in single wards using deterministic approaches [13,16]. As the population size in a single ward or hospital is likely to be small, a stochastic modelling approach may often be more appropriate as it can take account of the role of chance in determining transmission patterns. In Table 2, a definition of the modelling terms used for model classification is provided. Figure 5a shows that the proportion of stochastic models has increased steadily over time, and as Figure 6 illustrates, stochasticity was soon introduced (in 1997) [77] after publication of the first (deterministic) HCAI model. Several studies developed both a stochastic and a deterministic version of a similar compartmental model to investigate whether projected intervention effects were partly a result of random fluctuation [18,35,40,78-80]. Others use a deterministic model to interpret the findings of a stochastic model [81].

**Table 2:** Healthcare infection control interventions evaluated by a modelling framework (1997–2011)

**Pathogen**	**Interventions studied**	**First published**	**References**
**MRSA**	Hand hygiene	1997	[15-17,28,29,33,34,37,40,44-46]
	Antibiotic stewardship	1997	[16,21]
	Isolation	1997	[14,16,26,32,35,41,42,45]
	HCW cohorting	2002	[17,29,40,44,45]
	Screening	2005	[14,23,25,32,34,39,44,45]
	Decolonisation	2009	[14,25,26,33,34,40,45,46]
	Patient cohorting	2007	[40]
	Gown and glove use	2009	[32]
	Other	2006	[43]
**VRE**	Hand hygiene	1998	[12,21,28,47,49,51,54,55]
	Antibiotic stewardship	1999	[47,51,55]
	Isolation	2004	[12,52]
	HCW cohorting	1998	[12,49,51,54,55]
	Screening	2004	[47,52]
	Decolonisation	2007	[50]
	Patient cohorting	2008	[47]
	Environmental cleaning	2008	[47]
***C. difficile***	Other	2009	[59]
**ARB**	Hand hygiene	1997	[82]
	Antibiotic stewardship	1997	[67,78,83-88]
	Barrier precautions (i.e. not specified)	2000	[85]
**HCAI in general**	Hand hygiene	1999	[89,90]
	Isolation	2005	[77,91]
	HCW cohorting	2006	[77,90]
	Screening	1999	[89]
	Vaccination	2008	[77]
	Barrier precautions (i.e. not specified)	2007	[79]
	Patient cohorting	2005	[91,92]
	Environmental cleaning	2007	[92]
	Antibiotic prophylaxis	2007	[79]
	Antibiotic stewardship	2008	[93]
	HCW cohorting	2005	[91]
**HIV**	Sterilization of medical appliances	1999	[94]
**Influenza or ILI**	Vaccination	2008	[95-97]
	Prophylaxis	2009	[98]
	Other	2008	[99,100]
**Pertussis**	Vaccination	2009	[72,101]
**Rotavirus**	Hand hygiene	2011	[81]
	HCW cohorting	2011	[81]
	Vaccination	2011	[81]
**SARS**	Isolation	2007	[102]
	Barrier precautions (i.e. not specified)	2005	[74]
**TB**	Isolation	2007	[75]
	HIV treatment	2007	[75]
	Air ventilation	2007	[75]
	Facial mask	2007	[75]

#### Compartmental vs. individual-based

Infectious disease models can have either an aggregate (or compartmental) structure (which tracks groups in the population) or an individual-based structure (which tracks individuals). The latter enables better incorporation of heterogeneity in patient characteristics such as patient demographics, contact patterns and disease history, but at the cost of increased computational burden. To date, most (73%; 70 studies) HCAI models have taken an aggregate approach, although the proportion of individual-based models has increased over time (Figure 5a). In total, 26 publications (27%) took an individual-based approach of which seven papers (8%) used both compartmental and individual-based modelling [25,34,60,72,83,95,96].

#### Model fitting to data

Model parameter values can be based on existing studies, assumptions, or estimated directly from data [103]. Unknown parameters, such as infection transmission rates, can be inferred by calibrating a model to empirical data. With the increasing availability of computational power, numerically-intensive statistical methods for parameter inference have become more accessible. As Figure 5b shows, although only 35% (34 studies) of HCAIs models have incorporated some sort of calibration process to empirical data, this proportion has increased over time. Metrics used to quantify goodness of fit include the least square criterion (minimisation of sums of squares between the observed data and the model predictions) [21,56,57,75], maximum likelihood estimation (identification of the parameter value(s) that makes the observed data most likely) [18,22,24,35,53,63,65,66] and since 2007, Bayesian methods; frequently using Markov Chain Monte Carlo (MCMC) approaches [19,32,40,41,50,58,64,76] or a combination of MCMC and maximum likelihood estimation [36,59]. A further seven studies reported fitting their models by comparing model predictions to observed epidemiological data but did not apply any formal quantitative approach [17,29,43,60,81,101,104].

#### Uncertainty in model predictions

Infectious disease models are developed and informed using a combination of available evidence, for example on infection transmission, disease natural history and intervention effectiveness. As availability of such information is unlikely to be complete, mathematical models inherently include some degree of uncertainty. This uncertainty may relate to model parameter values, model structure (e.g. in terms of disease states incorporated and the relationship between them) or methodology used [9,105].

Parameter uncertainty was investigated by 36% of the studies (35 publications). As Figure 5b illustrates, similar trends as seen for the application of formal model calibration apply for the inclusion of parameter uncertainty. Also the methods used for parameter uncertainty have become more complex over time (Figure 5c). Of the 35 studies that have investigated parameter uncertainty, univariate sensitivity analysis (i.e. alteration of one parameter at a time whilst holding others at their base-case value) was conducted by 43% (15 studies) [18,28,29,43,44,46,60,63],[69,77,81,83,89,91,99]. The more computationally expensive probabilistic sensitivity analysis (formulation of uncertainty in the model inputs by a joint probability distribution, and propagating this uncertainty to the outputs [106]) is in general considered a rigorous method to account for uncertainty in the joint distribution of the parameters. This was employed by 51% (18 studies) [14,32,36,40-42,48,50,57-59,64],[75,76,78,95,96,98] among which Latin Hypercube Sampling (LHS) as a means of performing probabilistic sensitivity analysis was conducted by four studies [75,95,96,98]. Probabilistic sensitivity analysis utilizing LHS provides a rigorous method of incorporating and representing real uncertainty surrounding parameter estimates into model-based analysis where joint probability distributions for parameters are available.

#### Model validation

Model validation is rare in HCAI modelling. Ideally, a model should be validated by means of comparing the model predictions with observations from an alternative dataset than the one used for model fitting, although this is often difficult in practice. Four studies (5%) reported some kind of model validation based on at least two different data sets [50,53,75,101]. However, only one study used a statistical approach [101], whereas the others included subjective comparison of the model predictions (on infection transmission) with genotype data [50,53,75].

#### Setting and interaction between settings

Mathematical models of HCAIs have primarily been set in a single ward (49%, 47 studies), with the intensive care unit (ICU) being the most frequent setting modelled (26%, 25 studies) [14,16,22,28,29,31,32,36],[40-42,45,49,52,53,55,63,65],[72,79,82,91,101,107,108] or a simplified hospital setting, lacking any further ward structure (31%, 30 studies) [12,13,24,27,33,34,38,39],[45,46,51,58,60,64,66,68],[69,74,77,78,83-88,93,94,97],[109]. More recent studies however, have incorporated the interaction between general wards and the ICU [23,43,69] or between different wards [11]. Although these ward or hospital-based models do not usually treat the hospital as a closed system (i.e. hospital admission and discharge rates from and to a 'general community' are frequently included), transfer patterns between healthcare institutes are rarely considered [19,20,25,70,71,73], as are transmission dynamics within settings outside the healthcare facilities. The interaction between community and hospital transmission has been included for MRSA [30,35], antimicrobial resistant bacteria as a whole [67], Severe Acute Respiratory Syndrome [76,102] and tuberculosis [75]. Hence any possible long-term feedback between the hospital and other settings is not taken into account. Only two models concerned nosocomial transmission in a LTCF setting alone, i.e. of influenza [98] and norovirus [104] respectively.

## Discussion

Models of MRSA transmission dominate the literature, followed by VRE, although to a considerably lesser extent. Both have been the subject of national surveillance and infection control policies in a variety of developed countries [110-112]. This may account for the relative abundance of modelling studies*.* Despite causing a high burden and being the subject of national control policies [113,114], *C. difficile* transmission has seldom been modelled. Similarly, bloodstream infections due to third-generation cephalosporin-resistant *E. coli,* which have been estimated to cause ~2,700 excess deaths and 120,000 extra bed days in Europe in 2007 have been considered by only one study [65]. For comparison, MRSA was estimated to cause ~5,500 deaths and 256,000 additional bed days in Europe [115], yet has been the subject of over 30 studies. It seems then that the occurrence of models does not necessarily correlate to the burden of disease. This is also true in low and middle income countries, where a recently published systematic review [116,117] demonstrated significantly higher prevalence of HCAIs than in high income countries; however, very few modelling studies have tackled the problems of HCAI in less developed settings.

In terms of model methods, considerable changes can be identified over time. After the introduction of the first deterministic HCAI modelling study, inclusion of stochasticity has become common practice. The majority of the HCAI models evaluate infection control policies, for which sound model parameterisation and sensitivity analyses are required for reliable predictions. The use of more sophisticated methods for model parameterisation (e.g. MCMC) and uncertainty analysis has become increasingly common.

HCAI models have also increased in complexity regarding the settings modelled. Although the majority of the models have considered a single ward (often ICUs), the apparent emergence of transmission of typical HCAIs in the community, in particular of MRSA [118]*,* have resulted in models which consider the transmission of HCAI from a more holistic approach. As the long-term feedback loop related to hospital discharge and readmission of colonised patients and spread of HCAI pathogens in the community or settings as LTCFs can effect HCAI transmission dynamics [19,70,119], such an approach can aid in providing a realistic estimate of existing and new infection control strategies’ effectiveness.

This review has some limitations. First of all we have exclusively considered peer-reviewed publications in English. This might have resulted in a slight inaccuracy in our results, e.g. with regards to the modelling of particular pathogens in alternative national settings. We were exclusively interested in models exploring the patient-to-patient transmission of HCAI and antimicrobial resistance within healthcare settings (either directly, or mediated by healthcare workers and/or the healthcare environment). This has resulted in the exclusion of a higher number of models that elucidate the dynamics of antimicrobial resistance in its own right, which are summarised elsewhere [120,121]. Moreover, this review intended to provide overall trends in the field of HCAI modelling, rather than a detailed account of the quality of individual models and of what these models have shown, which could be a valid future area of investigation.

Compartmental models (which group individuals in classes) have predominated the field of HCAI modelling. The emergence of individual-based modelling allows for more realistic modelling of healthcare worker-patient contact (e.g. super spreading events) or incorporation of heterogeneity in transmission risk profiles of patients. However, these approaches are computationally far more intensive, are difficult to fit to data, and the inclusion of additional factors makes more demand on the data available. Detailed level data such as observed healthcare worker-patient contact collected for example via mote-based sensor networks, as has been done recently [122], could help parameterise such more complex models.

Moreover, recent technological developments in microbiology have resulted in enhanced access to pathogen sequence data, which could help to further improve HCAI models. Such data are beginning to inform disease outbreaks e.g. of avian influenza A (H7N7) [123] and Foot-and-Mouth disease [124]. Importantly, the increasingly routine use of sequencing of genetic material for epidemiological purposes can provide valuable insight, such as aiding in the understanding of the role of asymptomatic carriers in transmission (e.g. of *C. difficile*) and evolution of antimicrobial resistance.

## Conclusions

Transmission models concerning HCAI have showed a general enhancement in complexity, but have been almost completely limited to high-income settings, and have strongly focused on MRSA transmission in hospital settings. Further improvements in the availability of data and statistical methods could enhance the insight gained from these models.

## Abbreviations

ARB: Antimicrobial resistant bacteria; CRE: Cephalosporin-resistant Enterobacteriaceae; ESBL: Extended-spectrum beta-lactamases; HCAI: Healthcare-associated infections; ICU: Intensive care unit; ILI: Influenza-like illness; LHS: Latin Hybercube Sampling; LTCF: Long-term care facility; MCMC: Markov Chain Monte Carlo; MRSA: Methicillin-resistant *Staphylococcus aureus*; R-GNR: Third generation cephalosporin-resistant Gram-negative rods; TB: Tuberculosis; VRE: Vancomycin-resistant Enterococcus.

## Competing interests

The authors declare that they have no competing interests.

## Authors’ contributions

EvK developed the search strategy in collaboration with JR, and conducted a title-abstract screening, independent from a shared title-abstract screening by MJ, SD and WJE. Full text evaluation was conducted by EvK and in case of uncertainty, discussion took place with JR. EvK wrote the manuscript with significant contributions from the other authors. All authors have read and approved the final manuscript.

## Pre-publication history

The pre-publication history for this paper can be accessed here:

http://www.biomedcentral.com/1471-2334/13/294/prepub

## Supplementary Material

Additional file 1 Search terms MEDLINE. Click here for file
