# Supplementary material for: Modelling the transmission of healthcare associated infections: a systematic review
Source: BMC Infect Dis. 2013 Jun 28;13:294. doi: 10.1186/1471-2334-13-294 (PMC3701468; doi:10.1186/1471-2334-13-294)
Supplement: Additional file 1 — Search terms MEDLINE. [file 1471-2334-13-294-S1.docx]

# **Additional file 1: Search terms MEDLINE**

The search strategy was identical for EMBASE, Scopus, Global Health and CINHAL plus except for the MESH terms used, which were adjusted (or absent) for each database of concern.

ti,ab = Search for specified search term in title, abstract

mp = Search for specified search term in title, abstract, subject heading, heading word, drug

trade name, original title, device manufacturer, drug manufacturer, device trade name and

keyword

/ = MESH/EMTREE term

$ = Truncation

adj(N) = The maximum number of words allowed between the specified search terms

1. clostridium.ti,ab.

2. CDI.ti,ab.

3. CDAD.ti,ab.

4. VRSA.ti,ab.

5. VISA.ti,ab.

6. MSSA.ti,ab.

7. MRSA.ti,ab.

8. staphylococc$.ti,ab.

9. Streptococc$.ti,ab.

10. acinetobacter.ti,ab.

11. klebsiella.ti,ab.

12. Enterococc$.ti,ab.

13. Escherichia.ti,ab.

14. E Coli.ti,ab.

15. Enterobacter$.ti,ab.

16. citrobacter.ti,ab.

17. serratia.ti,ab.

18. Burkholderia.ti,ab.

19. Pseudomonas.ti,ab.

20. proteus.ti,ab.

21. Chryseobacteri$.ti,ab.

22. Flavobacteri$.ti,ab.

23. Alcaligenes.ti,ab.

24. Achromobacter.ti,ab.

25. legionell$.ti,ab.

26. Mycobacteri$.ti,ab.

27. rotavirus.ti,ab.

28. norovirus.ti,ab.

29. Respiratory Syncytial Viruses.ti,ab.

30. Hepatitis.ti,ab.

31. ebola.ti,ab.

32. Varicella-zoster.ti,ab.

33. Cytomegalovirus.ti,ab.

34. Adenovirus.ti,ab.

35. Giardia lamblia.ti,ab.

36. Candida albicans.ti,ab.

37. Aspergillus.ti,ab.

38. Cryptococc$.ti,ab.

39. Cryptosporidi$.ti,ab.

40. herpes$.ti,ab.

41. SARS.ti,ab.

42. Severe Acute Respiratory Syndrome.ti,ab.

43. Influenza.ti,ab.

44. Microbial-drug-resistan$.ti,ab.

45. Antibiotic-resistan$.ti,ab.

46. Antimicrobial-resistan$.ti,ab.

47. Multidrug resistan$.ti,ab.

48. **or/1-47**

49. hospital$.ti,ab.

50. nosocomial.ti,ab.

51. healthcare.ti,ab.

52. health care.ti,ab.

53. exp hospital/

54. exp hospital units/

55. **or/49-54**

56. **48 and 55**

57. exp Clostridium/

58. Clostridium difficile/

59. exp Clostridium Infections/

60. exp Staphylococcus aureus/

61. exp Staphylococcal Infections/

62. exp Escherichia coli/

63. exp Escherichia coli Infections/

64. exp Streptococcus/

65. exp Streptococcal Infections/

66. exp Klebsiella/

67. exp Klebsiella Infections/

68. exp Acinetobacter/

69. exp Acinetobacter Infections/

70. exp Enterobacter/

71. exp Citrobacter/

72. exp Serratia/

73. exp Serratia Infections/

74. exp Enterococcus/

75. exp Burkholderia/

76. exp Pseudomonas/

77. exp Burkholderia Infections/

78. exp Pseudomonas Infections/

79. exp Proteus/

80. exp Proteus Infections/

81. exp Flavobacteriaceae/

82. exp Alcaligenes/

83. exp Achromobacter/

84. exp Legionella/

85. exp Mycobacterium/

86. exp Rotavirus/

87. Rotavirus Infections/

88. exp Norovirus/

89. exp Respiratory Syncytial Viruses/

90. Influenza, Human/

91. exp Hepatitis, Viral, Human/

92. exp Enterovirus B, Human/

93. exp Enterovirus/

94. Enterovirus Infections/

95. exp Herpesviridae/

96. Ebolavirus/

97. exp Adenoviridae/

98. exp Giardia/

99. SARS Virus/

100. Severe Acute Respiratory Syndrome/

101. Candida albicans/

102. exp Aspergillus/

103. exp Cryptococcus/

104. exp Sarcoptes scabiei/

105. exp Drug Resistance, Microbial/

106. **or/57-105**

107. **48 or 106**

108. **55 and 107**

109. exp Cross Infection/

110. Infectious Disease Transmission, Professional-to-Patient/

111. Infectious Disease Transmission, Patient-to-Professional/

112. Cross infection.mp.

113. (professional-to-patient adj2 tranmission).mp.

114. (patient-to-professional adj2 transmission).mp.

115. (patient-to-patient adj2 transmission).mp.

116. (Healthcare-associated adj2 infect$).mp.

117. (Healthcare-associated adj2 disease$).mp.

118. (Hospital-acquired adj2 infect$).mp.

119. (Hospital-acquired adj2 disease$).mp.

120. (Hospital-onset adj2 infect$).mp.

121. (Hospital-onset adj2 disease$).mp.

122. (Nosocomial adj2 infect$).mp.

123. (Nosocomial adj2 disease$).mp.

124. (Hospital adj2 transmiss$).mp.

125. (Hospital adj2 infect$).mp.

126. HCAI.mp.

127. HAI.mp.

128. **or/109-127**

129. Mathematic$.ti,ab.

130. Compartment$.ti,ab.

131. Stochastic.ti,ab.

132. Deterministic.ti,ab.

133. transmiss$.ti,ab.

134. Epidemi$.ti,ab.

135. Individual-based.ti,ab.

136. Population-based.ti,ab.

137. dynamic.ti,ab.

138. or/129-137

139. Model$.ti,ab.

140. Model?ing.ti,ab.

141. Framework$.ti,ab.

142. or/139-141

143. 138 and 142

144. Models, Theoretical/

145. mathematical computing/

146. Basic Reproduction Number/

147. Basic reproduction number.mp.

148. Effective reproduction number.mp.

149. Computer Simulation/

150. Markov chains/

151. Monte Carlo Method/

152. Bayes Theorem/

153. exp Stochastic Processes/

154. **or/144-153**

155. ((Mathematic$ or Compartment$ or Stochastic or Deterministic or Transmission or Epidemi$ or Individual-based or population-based or Markov or Bayesian or equation or theoretic$ or cost-effective$ or cost-benefit or cost-consequence$ or $economic$ or discrete-event or micro or agent-based or decision or decision-analytic or decision-tree) adj5 (Model$ or Model?ing or Framework$ or simulation$)).ti,ab.

156. **108 or 128**

157. **154 or 155**

158. **156 and 157**
